# Suggestions to Soldiers

**Published:** 1861-11

**Authors:** 


IT IS SUGGESTED that soldiers be instructed to micturate, before going into battle, with a view of lessening, by the diminished size of the empty organ, the chances of wounds of that viscus, and the consequent frequent and fatal complcation—peritoneal unrinary extravasation.

